# PCSK9 loss-of-function variants and risk of infection and sepsis in the Reasons for Geographic and Racial Differences in Stroke (REGARDS) cohort

**DOI:** 10.1371/journal.pone.0210808

**Published:** 2019-02-06

**Authors:** Kellie A. Mitchell, Justin Xavier Moore, Robert S. Rosenson, Ryan Irvin, Faheem W. Guirgis, Nathan Shapiro, Monika Safford, Henry E. Wang

**Affiliations:** 1 University of Alabama School of Medicine, Birmingham, Alabama, United States of America; 2 Department of Epidemiology, University of Alabama at Birmingham, Birmingham, Alabama, United States of America; 3 Department of Emergency Medicine, University of Alabama School of Medicine, Birmingham, Alabama, United States of America; 4 Mount Sinai Heart, Icahn School of Medicine at Mount Sinai, New York, New York, United States of America; 5 Department of Emergency Medicine, University of Florida Jacksonville, Jacksonville, Florida, United States of America; 6 Department of Emergency Medicine, Beth Israel Deaconess Medical Center, Boston, Massachusetts, United States of America; 7 Department of Medicine, Weill-Cornell School of Medicine, New York, New York, United States of America; 8 Department of Emergency Medicine, University of Texas Health Science Center at Houston, Houston, Texas, United States of America; International University of Health and Welfare, School of Medicine, JAPAN

## Abstract

**Background:**

Elevated proprotein convertase subtilisin/kexin type 9 (PCSK9) levels have been associated with adverse outcomes in patients hospitalized for sepsis. PCSK9 loss-of-function (LOF) variants area associated with lower low-density lipoprotein cholesterol (LDL-C) levels. Decreased LDL-C is a biomarker of acute and chronic infection and sepsis risk. We examined the association between presence of two genetic PCSK9 LOF variants and risk of infection and sepsis in community-dwelling adults.

**Methods:**

We analyzed data from 10,924 Black participants tested for PCSK9 LOF variants in the REasons for Geographic and Racial Differences in Stroke (REGARDS) cohort. The primary endpoint was hospitalization for a serious infection. Within serious infection hospitalizations, we defined sepsis as ≥2 system inflammatory response syndrome criteria. Using multivariable Cox and logistic regression, we investigated the association between LOF variants and hospitalization for infection and sepsis events, adjusting for sociodemographics, health behaviors, chronic medical conditions and select biomarkers.

**Results:**

Among 10,924 Black participants, PCSK9 LOF variants were present in 244 (2.2%). Serious infection hospitalizations occurred in 779 participants (14 with PCSK9 variants and 765 without). The presence of PCSK9 variants was not associated with infection risk (adjusted HR 0.68; 95% CI: 0.38–1.25). Among participants hospitalized for a serious infection, the presence of PCSK9 variants was not associated with sepsis (adjusted OR 7.31; 95% CI = 0.91–58.7).

**Conclusions:**

PCSK9 LOF variants are not associated with increased risk of hospitalization for a serious infection. Among those hospitalized for a serious infection, PCSK9 LOF variants was not associated with odds of sepsis.

## Introduction

Sepsis is the syndrome of infection complicated by systemic inflammation and organ dysfunction. Low levels of low density lipoprotein cholesterol (LDL-C), a negative acute phase reactant, have been associated with organ failure and mortality in sepsis. Multiple clinical observations have displayed an association linking lower cholesterol levels with higher sepsis severity and mortality.[1–3] Moreover, in a previous study of the population-based REGARDS cohort we found an association between baseline low LDL-C and higher rates of future sepsis events.[4]

Proprotein convertase subtilisin/kexin type 9 (PCSK9) is a zymogen in the proprotein convertase family involved in the regulation of hepatic LDL receptors (LDL-Rs) and therefore affects LDL and LDL-C clearance. A single gene produces PCSK9, which autocleaves in the endoplasmic reticulum to a mature form that, upon release from cells, can bind LDL-Rs for endocytosis and lysosomal degradation; PCSK9 loss-of-function (LOF) variants result in no autocleavage of the protein, and the protein is not able to bind LDL-Rs. [5] Therefore, humans with a PCSK9 loss-of-function variant have increased LDL-R activity and higher LDL-C clearance rates. Three nonsense mutations have been shown to have significant effects on LDL-C levels: R46L, which is more common in Whites, and Y142X and C679X, which are more common in Blacks–other variants in PCSK9 do not tend to show consistent, significant reductions in LDL-C.[6] Overall, about 2% of Blacks have Y142X or C679X, which results in LDL-C levels that are approximately 40% lower than controls, while the R46L variant in Whites is associated with 21% lower LDL-C compared to controls.[7]

Given the associations between PCSK9 and LDL-C, and then LDL-C and immune response, there are plausible connections between PCSK9 variants and risk of hospitalization for serious infections and sepsis. For example, PCSK9’s LDL-R degrading function may decrease hepatocyte endotoxin clearance.[8] A murine study found that PCSK9 inhibition reduced sepsis mortality.[9] PCSK9 levels have been reported to be increased in septic patients, leading to decreased endotoxin clearance and increased rates of organ failure.[10] Walley et al. found better outcomes of septic shock in patients with lower serum PCSK9 protein levels.[11]

In this study, we sought to determine the association between the presence of PCSK9 LOF variants and risk of hospitalizations for community-dwelling Black adults. Among first-infection hospitalizations, we also sought to determine if PCSK9 LOF variants are associated with odds of sepsis. Given that our prior REGARDS investigation observed that lower serum LDL-C levels were associated with higher risk of sepsis and that a PCSK9 LOF variant results in lower serum LDL-C, we hypothesized that PCSK9 LOF variants would be associated with increased risk of developing future serious infections and sepsis.

## Methods

### Study design

We utilized data from the REasons for Geographic and Racial Differences in Stroke (REGARDS) study cohort. The Institutional Review Board of the University of Alabama at Birmingham approved this study.

### Data source—The REGARDS cohort

The REGARDS cohort is an ongoing longitudinal cohort of 30,239 community-dwelling adults in the United States.[12] REGARDS recruited White and Black adults 45 years of age or older during January 2003 through October 2007. [12] Approximately 55% of the cohort is female, 41% is Black, and 69% is aged 60 years and older. Baseline information collected from each participant included demographic, health behaviors, medical history, diet and exercise habits and medications. During baseline interview, REGARDS also collected blood and urine samples from each participant. [12] REGARDS contacted participants every six months to inquire about recent hospitalizations and illnesses. In the case of participant death, REGARDS interviewed proxies and conducted reviews of death certificates and medical records of the deceased in order to accurately document the cause of death. [12]

### Identification of PCSK9 loss-of-function variants

We determined the presence of the PCSK9 variant using the Gentra Puregene Blood kit (Qiagen #158389). Extracted DNA was stored in aliquots at 4°C and -80°C. Analysis of PCSK9 variant presence focused on two nonsense variants in Blacks: Y142X (rs67608943) and C679X (rs28362286). We limited the analysis to Blacks because PCSK9 LOF nonsense variants in Whites do not have the same level of association with LDL-C levels as they do in Blacks (about 21% decrease in LDL-C in Whites versus about 40% decrease in Blacks).[7] Also, PCSK9 LOF variant status was known for fewer than 2,000 Whites in the REGARDS cohort.

### Identification of serious infection and sepsis events

The primary outcomes of the study were 1) first serious infection hospitalization, and 2) among first serious infection hospitalizations, the presence of sepsis. We used hospital admission and Emergency Department records to identify hospitalizations attributed to a serious infection.[13] For each hospitalization event, two abstractors independently reviewed all relevant medical records to verify the presence of serious infection at admission and infection as a primary reason for hospitalization. Abstractor agreement and additional physician-level review resolved any discordances.

Among first infection hospitalizations, we defined sepsis events as participants with a minimum of two Systemic Inflammatory Response Syndrome (SIRS) criteria that included 1) fever (temperature >38.3°C or <36°C), 2) heart rate >90 beats per minute, 3) tachypnea (>20 breaths per minute) or PCO2<32 mmHg, and 4) leukocytosis (white blood cell count >12,000 or <4,000 cells/mm^3^ or >10% band forms).[14] The diagnosis of SIRS was established from tests and vital signs obtained during the first 28 hours of hospitalization. In order to maintain consistency with prior REGARDS-sepsis analyses, we did not include organ dysfunction in the definition of sepsis.

We included hospitalization events reported between January 1, 2003 and December 31, 2012.

### Participant demographics

Participant demographics assessed in the analysis include sociodemographics, health behaviors, chronic medical conditions, and select biomarkers. (Table 1) We identified alcohol intake according to the National Institute on Alcohol Abuse and Alcoholism classification.[15] We classified smoking status as smokers, past smokers, or current smokers. We observed presence of the following comorbidities: atrial fibrillation, chronic lung disease, coronary artery disease, deep vein thrombosis (self-reported), diabetes mellitus, chronic kidney disease, dyslipidemia, hypertension, obesity, stroke history (self-reported), and peripheral artery disease (self-reported). Dyslipidemia was defined as an LDL-C level > 130 mg/dL in accordance with guidelines from the American Association of Clinical Endocrinologists,[16] or participant reported use of lipid-lowering medications. We used biomarkers associated with lipid levels including LDL-C, HDL-C, serum total cholesterol, and triglycerides. HDL-C, serum total cholesterol, and triglycerides were directly measured from serum samples whereas LDL-C was calculated using the Friedewald formula from total cholesterol, HDL-C, and triglycerides. Other biomarkers included cystatin C and high sensitivity C-reactive protein.[17, 18]

**Table 1:** Baseline participant characteristics, stratified by presence or absence of a PCSK9 loss-of-function variant. Includes 10,924 Black REGARDS participants.

Characteristic	PCSK9 Variant Status		
	Present (n = 244)	Absent (n = 10,680)	*P* value
**Age (yrs)–mean (SD)**	64 (9.1)	64 (9.2)	.52
**Gender (%)**			.93
Male	38.1	38.4	
Female	61.9	61.6	
**Education (%)**			.10
Less than High School	6.5	8.7	
High School	28.3	22.8	
Some College	32.6	29.6	
College or more	32.6	38.9	
**Income (%)**			.31
<$20k	30.3	26.4	
$20k-$34k	28.3	26.4	
$35-$74k	22.1	25.8	
≥$75k	6.6	9.2	
Not Available	12.7	12.3	
**Geographic Region (%)**			.92
Non-Stroke Belt or Buckle	48.8	48.9	
Stroke Belt	34.4	33.5	
Stroke Buckle	16.8	17.7	
**Alcohol Use (%)**			.36
None	76.1	72.0	
Moderate	21.4	25.5	
Heavy	2.6	2.5	
**Smoking (%)**			.18
Never	47.7	45.3	
Past	32.1	37.5	
Current	20.2	17.2	
**Comorbidities (%)**			
Atrial Fibrillation	5.9	7.8	.27
Chronic Lung Disease	9.8	7.8	.23
Coronary Artery Disease	11.2	15.6	.07
Deep Vein Thrombosis	5.8	4.9	.55
Diabetes Mellitus	34.0	30.5	.23
Chronic Kidney Disease	13.5	12.1	.49
Dyslipidemia	26.2	54.3	< .001
Hypertension	70.1	71.4	.66
Obesity	62.1	62.8	.85
Stroke	7.8	4.9	.09
Peripheral Artery Disease	2.5	2.4	.93
**Biomarkers**			
LDL Cholesterol mg/dL (mean, SD)	84.8 (32.0)	117 (36.2)	< .001
HDL Cholesterol mg/dL (mean, SD)	53.8 (16.0)	53.4 (16.0)	.73
Total Cholesterol mg/dL (mean, SD)	161 (35.1)	194 (41.0)	< .001
Triglycerides mg/dL (mean, SD)	111 (58.3)	113 (73.5)	.59
Estimated Glomerular Filtration Rate mL/min/1.73 m^2^ (mean, SD)	87.8 (24.5)	88.4 (23.6)	.73
Cystatin C >1.12 mg/dL (%)	23.7	27.8	.16
hsC-reactive protein >3.0 mg/dL (%)	45.5	48.3	.38
**Lipid Lowering Medication Use**			
Cholestyramine	0.0	0.1	.69
Ezetimibe	0.8	2.6	.08
Fibrates	0.0	1.0	.12
Statins	13.1	29.5	<0.001

Hospital course variables consisted of Sequential Organ Failure Assessment (SOFA) scores for coagulation, central nervous system, and respiratory, renal, cardiovascular, and liver systems, culminating in a total SOFA score, and Mortality in Emergency Department Sepsis (MEDS) scores. We also noted the type of infection and intensive care unit (ICU) admission.

### Data analysis

We determined differences in participant characteristics between those with and without PCSK9 variants using Chi-square tests for categorical variables, t-tests for parametric continuous variables, and Wilcoxon rank-sum tests for non-parametric continuous variables. To determine the independent association of PCSK9 variant presence with incident serious infection, we fit a series of Cox proportional hazards models, adjusting for sociodemographics, health behaviors, comorbid medical conditions, and biomarkers. We excluded dyslipidemia, LDL-C levels, and total cholesterol levels from these models because they are part of the PCSK9 pathway. To explore an alternate risk adjustment strategy, we fit an additional model adjusted for the REGARDS Sepsis Risk Score (SRS). The SRS is a previously derived scale characterizing 10-year risk of sepsis based upon the several biomarkers, sociodemographics, and health behaviors, and it uses these factors to define five categories of risk severity.[19]

Among first infection events, we performed logistic regression to examine the association between PCSK9 variant presence or absence and sepsis adjusted for sociodemographics, health behaviors, comorbidities, biomarkers, and 28-hour Sequential Organ Failure Assessment (SOFA) score. We also fit an alternate model adjusted for the SRS categories and SOFA score. We performed all analyses using Stata 14.1 (Stata, Inc., College Station, Texas).

## Results

Among 12,514 Black participants, 10,934 consented to genetic testing, and 10,924 completed testing. Of these, 190 tested positive for the C679X variant and 54 tested positive for the Y142X variant; the overall prevalence of any PCSK9 LOF variant was 2.2% (Table 1). There were no statistically significant differences in sociodemographic factors or health behaviors (alcohol and smoking) between those with and without PCSK9 variants. Participants with PCSK9 LOF variants were less likely to have dyslipidemia when compared with other participants (26.2% vs 54.3%, *P* < .001) and had lower total cholesterol levels (mean values 161 vs 194 mg/dL, *P* < .001). There were no other biomarker differences between participants with and without the PCSK9 variants. Participants with PCSK9 LOF variants were less likely to report statin use.

**Fig 1:**
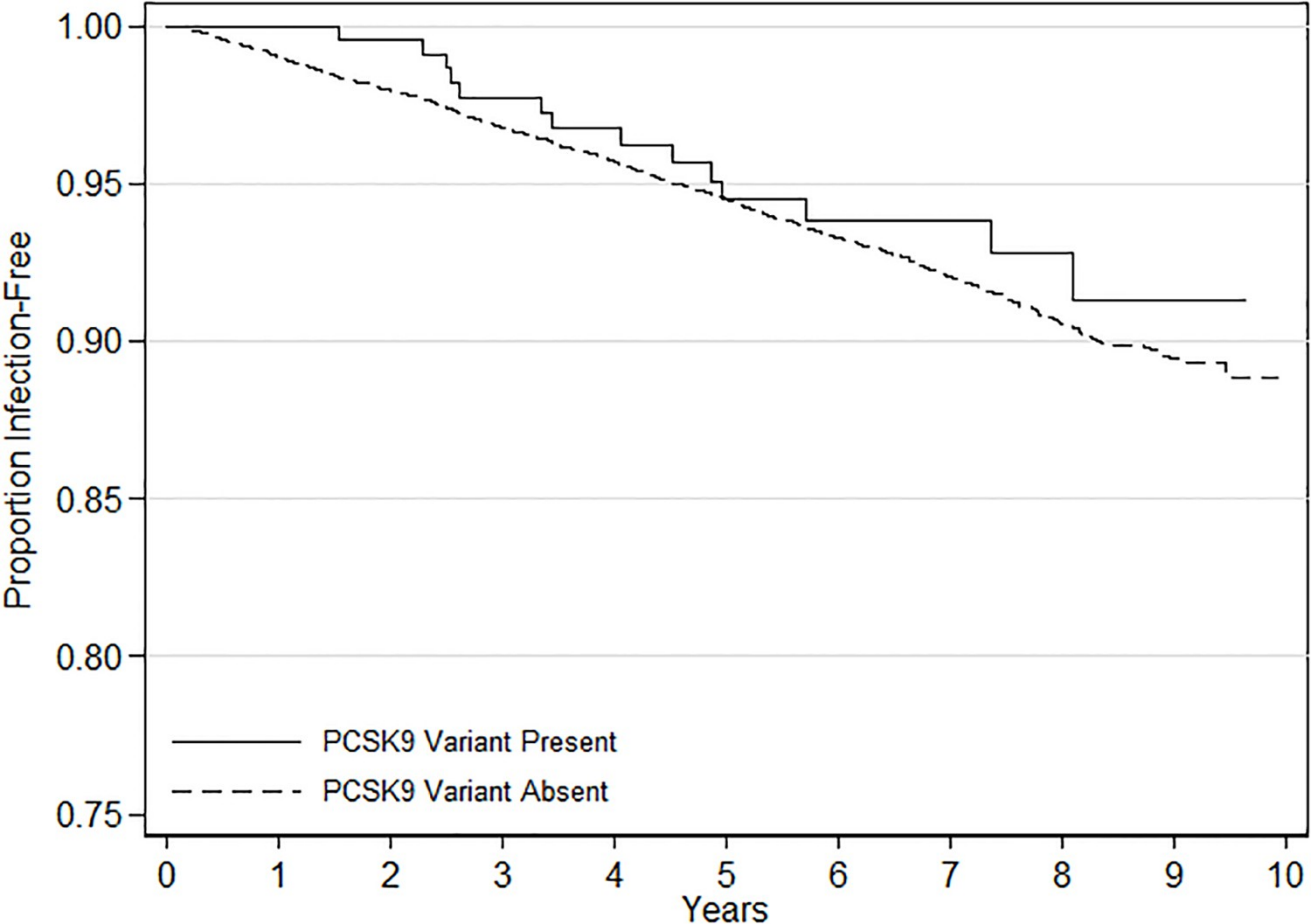
Kaplan-Meier survival curve for incident serious infection hospitalizations, stratified by presence or absence of PCSK9 variant. Analysis limited to blacks. Log-rank test p = 0.47.

There were a total of 779 hospitalizations for a serious infection, including 14 (5.74%) among participants with the variant and 765 (7.16%) among those without (Table 2, Fig 1). Respiratory infections were the most common type of serious infection. Hospital death, 30-day fatality, SOFA scores, and MEDS scores were similar between participants with and without a PCSK9 LOF variant. Participants with PCSK9 variants were more likely to be admitted to the intensive care unit (21.4% of infected variants vs. 7.3% of infected non-variants, p = 0.048). Among first infection hospitalizations, 447 (57.4%) met sepsis criteria. Among 367 lung infections, 217 (59.1%) met sepsis criteria. Among 169 kidney infections, 86 (51.2%) met sepsis criteria. Among 143 intrabdominal infections, 60 (45.5%) met sepsis criteria.

**Table 2:** Infection type and hospital course among n = 779 first serious infection hospitalizations. SOFA = Sequential Organ Failure Assessment. MEDS = Mortality in Emergency Department Sepsis. ICU-intensive care unit.

Variable	PCSK9 variant present (n = 14)	PCSK9 variant absent (n = 765)	*P* value
**Infection Type (n = 779)**			< .001
Lung	7 (50.0%)	311 (40.7%)	
Kidney	2 (14.3%)	150 (19.6%)	
Abdominal	0 (0.0%)	120 (15.7%)	
Skin	1 (7.1%)	102 (13.3%)	
Sepsis	4 (28.6%)	35 (4.6%)	
Other	0 (0.0%)	47 (6.1%)	
**Hospital Course Variables**			
SOFA Score (median, IQR)	1 (1–2)	1 (0–2)	.26
MEDS score (median, IQR)	11 (6–15)	9 (6–11)	.35
ICU admission (n = 779)	3 (21.4%)	56 (7.3%)	.05
**Outcomes**			
Sepsis Hospital Death	1 (7.1%)	46 (6.0%)	.86
30-day case fatality	4 (28.6%)	145 (19.0%)	.37

On multivariable analysis, there was no association between the presence of a PCSK9 variant and incident first serious infection; adjusted HR 0.68, 95% CI: 0.38–1.25 (Table 3). We observed similar associations after risk adjustment using the REGARDS Sepsis Risk Score. There were no associations with incident first infection when limited to the C679X variant (adjusted HR 0.79; 95% CI 0.42–1.48) or Y142X variant (0.30; 0.04–2.11). When limiting the analysis to first serious infection events, the presence of a PCSK9 LOF variant was not associated with odds of sepsis; adjusted OR 7.31 (95% CI: 0.91–58.7).

**Table 3:** Association of PCSK9 loss-of-function variants and risk of serious infection and sepsis hospitalizations. Serious infection risk estimated by Cox proportional hazards analysis. Odds of sepsis among serious infection hospitalizations estimated by logistic regression. CI = confidence interval, HR = hazard ratio, OR = odds ratio.

**Serious Infection** **Hospitalizations** **(N = 10,924)**	**No. Events**	**No. Non-Events**	**Events Per** **1,000 Person-Years** **(95%CI)**	**Crude HR** **(95% CI)**	**Model 1** **HR (95% CI)**	**Model 2** **HR (95% CI)**	**Model 3** **HR (95% CI)**
PCSK9 variant present	14 (5.7%)	230 (94.3%)	9.8 (5.8–16.5)	0.82 (0.48–1.40)	0.84 (0.49–1.42)	0.68 (0.38–1.25)	0.83 (0.49–1.40)
PCSK9 variant absent	765 (7.2%)	9,915 (92.8%)	11.9 (11.1–12.8)	Ref.	Ref.	Ref.	Ref.
Total	779 (7.1%)	10,145 (92.9%)	11.9 (11.1–12.7)	-	-	-	-
**Sepsis Within** **Serious Infection Hospitalizations** **(N = 779)**	**No. Events**	**No. Non-events**	**Events Per** **1,000 Person-Years** **(95%CI)**	**Crude OR** **(95% CI)**	**Model 1** **OR (95% CI)**	**Model 2** **OR (95% CI)**	**Model 3** **OR (95% CI)**
PCSK9 variant present	11 (2.5%)	3 (0.9%)	N/A	3.28 (0.91–11.84)	3.34 (0.91–12.19)	7.31 (0.91–58.7)	3.22 (0.89–11.6)
PCSK9 variant absent	404 (97.4%)	361 (99.2%)	N/A	Ref.	Ref.	Ref.	Ref.
Total	415 (53.3%)	364 (46.7%)	N/A	-	-	-	-

Model 1. Adjusted for demographics, smoking, and alcohol use

Model 2. Adjusted for model 1 + comorbidities + biomarkers

Model 3. Adjusted for sepsis risk score categories

## Discussion

In this study of 10,924 Blacks in the REGARDS cohort, we found no association between PCSK9 LOF variants and the incidence of serious infections. Among first infection hospitalizations, we also did not observe significant associations between the presence of PCSK9 LOF variants and odds of sepsis.

The literature linking PCSK9, LDL-C, and infection shows conflicting results. Some studies suggest that higher cholesterol and/or PCSK9 function are associated with better response to some infections[4, 20–23] while others reported lower risk and/or better outcomes with lower PCSK9 and/or LDL-C.[8–11, 24] It is important to consider the distinctions of these studies. Select studies involved murine models.[9] Walley, et al. demonstrated that LDL-R and PCSK9 are critical regulators of endotoxin clearance in sepsis.[8, 10, 11] However, Berger, et al. found that PCSK9 LOF mutations did not affect lipopolysaccharide-induced mortality and failed to find a role for LDL-R in the process at all. [25] Walley’s studies used human hepatic cells and only demonstrated increased hepatic clearance via LDL-R of endotoxin above a threshold level commonly present in human sepsis. In contrast, Berger, et al. used wild type mice or LDL-R-/- or Pcsk9+/- mice. This may be one reason for the discrepancy between these studies.

Lagrost, et al. evaluated total cholesterol without stratifying by LDL-C and HDL-C,[20] which is important given reported associations between HDL-C and infection and sepsis outcomes.[26, 27] LDL-C and PCSK9 have been reported to have a protective association against parasitic or viral infections, but most sepsis cases are thought to be due to bacterial pathogens.[28] Most importantly, many studies of PCSK9 and sepsis used circulating PCSK9 plasma levels gathered during sepsis events instead of at baseline.[1, 10, 11] Our study used genetic analysis to assess the presence of PCSK9 genetic variation, baseline LDL-C levels, and risk of future infection and sepsis events.

Our analysis conceptualized that the presence of a PCSK9 LOF variant would lead to decreased levels of PCSK9 protein, leading to decreased LDL-C and increased long-term rates of infection or sepsis. Our previous studies demonstrate that low cholesterol levels (specifically LDL-C) may increase sepsis risk.[4, 29] Barter, et al. found that CETP inhibitors lead to drastic elevations in HDL, reductions in LDL, and increased all-cause mortality.; death from infection comprised nearly half of the deaths.[30] However, the lack of an independent association between PCSK9 LOF variant presence and infection risk may suggest that an alternate pathway also likely links LDL-C with sepsis risk. For example, HDL binds endotoxin and/or lipotechoic acid from bacteria and returns it to the liver for elimination.[21] This process requires multiple steps, and perturbations in the pathway could include reduced HDL-C function or dysfunction, impairment in clearance via scavenger receptors, or other steps in the process. Moreover, during critical illness, impaired lipid absorption through the gut leads to lipolysis and increased triglyceride levels while cholesterol-containing lipoprotein levels plummet, with non-survivors having lower LDL-C, HDL-C, and lysophosphatidylcholine than survivors.[31] Therefore, the low LDL-C may be in part a symptom of impaired lipid absorption. Inflammation may also play a role in the connection between cholesterol and sepsis risk. In an acute inflammatory response, the composition of lipoproteins changes, increasing LDL oxidative susceptibility, fostering HDL proinflammatory dysfunction, and altering of the levels of both LDL-C and HDL-C.[32] If anti-inflammatory drugs are given, the lipid profile returns towards normal.[33] In addition, PCSK9 itself may act in inflammation. Some researchers observed PCSK9 induction in response to TNF-alpha, which has been implicated in chronic inflammation.[34] A recent study supports the role of PCSK9 in atherosclerotic inflammation, finding that PCSK9 promotes secretion of inflammatory cytokines in macrophages *in vitro* while *in vivo* PCSK9 silencing decreases vascular inflammation and reduces pro-inflammatory cytokine expression in apolipoprotein E knockout mice.[35] Ricci and colleagues found a similar effect in humans and report that PCSK9 influences induction of multiple pro-inflammatory cytokines.[36]

An important related question is whether PCSK9 inhibitors may influence infection risk. Two prior placebo-controlled Phase 3 studies reported minor infection as one of the most common adverse events in patients treated with the PCSK9 inhibitor Evolocumab.[37, 38] These findings contrast with our finding that PCSK9 LOF variants did not alter infection hospitalization risk. Assuming the validity of the prior studies, our current results suggest that a PCSK9 inhibitor may increase infection risk through mechanisms other than downregulation of hepatic LDL-Rs. For example, PCSK9 is expressed in other tissues including the intestine and kidneys, circulating PCSK9 does not uniformly affect LDL-Rs in all organ systems, and the ability of PCSK9 to bind to other receptors is still debated.[39] Proposed receptors include apolipoprotein E receptor 2, cluster of differentiation 36, LDL-R related protein 1, and very low-density lipoprotein receptor; these are present in various organs, including the central nervous system, hepatocytes, macrophages, adipocytes, and intestinal cells.[5] It is possible that PCSK9 variants and/or PCSK9 inhibitors interfere with these and other targets in addition to their effect on LDL-C, and that improperly cleaved PCSK9 variant proteins may have their own yet-undiscovered effects. Because much of the detail of PCSK9’s function beyond LDL-R downregulation is yet unknown, additional studies are needed to clarify the pathways of PCSK9 inhibitors and their effects on infection risk.

## Limitations

We limited the analysis to presence and absence of the Y142X or C679X variants in PCSK9 in Blacks. We did not assess other genomic variants. We did not assess these variants in Whites due to the emphasis of REGARDS on genetic risk in Blacks. REGARDS is not a surveillance study, and thus complete ascertainment of all infection or sepsis events is unlikely. By design, the REGARDS cohort includes only Blacks and Whites, and this study limited further only to Blacks. Therefore, these results may not generalize to other ethnic groups. There is potential for participation bias because some individuals did not consent to genetic testing. Infection events in REGARDS were based upon clinical documentation, not culture, laboratory or diagnostic imaging. We did not consider the causal organism of infection.

While we used multivariable adjustment to account for confounders, sample size was relatively modest, leading to some uncertain statistical inferences as evidenced by select odds ratios with wide confidence intervals. Because of the limited sample size, we opted not to further stratify the analysis by individual PCSK9 variant. While we had data on the presence or absence of chronic medical conditions, we did not have information on measures of severity. The focus of our study was on community-acquired sepsis, so we did not include cases of sepsis that occurred during hospital stay. The absence of an association between PCDK9 variants and cardiovascular disease risk was likely due to the limited sample size.

In our prior study using REGARDS data, we found no association between HDL-C and sepsis hospitalizations.[4] In contrast Madsen, et al. observed a U-shaped relationship between HDL-C and serious infection hospitalization in the Copenhagen General Population Study and the Copenhagen City Heart Study.[40] The latter study used a different cohort and identified infections using discharge diagnoses. In contrast, in REGARDS we identified sepsis event through structured review of clinical findings. The findings of the current analysis regarding PCKS9 variants and sepsis risk may differ if replicated in a different cohort.

## Conclusion

Among Blacks in the REGARDS cohort, we found no association between PCSK9 LOF variant presence and risk of serious infection hospitalization. Among first serious infection hospitalizations, there was no association between PCSK9 variant presence and the odds of sepsis. Other mechanisms may be responsible for links between LDL-C and sepsis risk.
